# A Conservative Approach to the Treatment of a Rare Case of Cervical Spine Double Expressor Diffuse Large B-cell Lymphoma: A Case Report

**DOI:** 10.7759/cureus.21208

**Published:** 2022-01-13

**Authors:** Wesley Chen, Busha Hika, Caitlyn J Smith, Timothy J Parrett, Fassil B Mesfin

**Affiliations:** 1 Neurological Surgery, University of Missouri School of Medicine, Columbia, USA; 2 Pathology and Anatomical Sciences, University of Missouri School of Medicine, Columbia, USA

**Keywords:** spinal lymphoma, double expressor spinal dlbcl, diffuse large b cell lymphoma, conservative approach, spinal tumours

## Abstract

Non-Hodgkin's lymphomas are a group of lymphoid neoplasms, with diffuse large B-cell lymphoma (DLBCL) being the most common subtype. Genetic alterations involving c-MYC, BCL-2, and BCL-6 have been implicated in the pathogenesis of subtypes of DLBCL with poor prognostic implications. This case report demonstrates a retropharyngeal mass with extension through the bilateral neuroforamina into the epidural space and posterior elements of the cervical spine (C2-C3), for which biopsy revealed diffuse large B-cell lymphoma. Here we present a unique case as it provides a solution for the dilemma on how to treat a patient with a known prior malignancy (gastrointestinal [GI] melanoma) with a retropharyngeal mass with epidural extension (dumbbell-shaped tumor) with an inconclusive initial CT-guided needle-core biopsy. A CT-guided biopsy only yielded that the mass was neoplasm; we had a choice between attempting gross total resection of the mass or open biopsy. Attempting gross total resection would have entailed an anterior approach (transoral with possible odontoidectomy or endoscopic endonasal with possible odontoidectomy) along with posterior instrumentation and fusion from occiput to C3, which is a rather morbid procedure that would subject the patient to a decreased quality of life as well as risks of vascular injury, dysphagia, and infection. We elected to perform an open biopsy of the epidural component of the mass through a decompressive laminectomy, which allowed for decompression of the spinal cord as well as a sampling of the mass. This provided treatment for possible increasing epidural compression from the mass, as well as diagnostic tissue. A multidisciplinary team discussed the case and developed a treatment plan for the patient with systemic and intrathecal chemotherapy in combination with radiotherapy.

## Introduction

Non-Hodgkin's lymphoma (NHL) is the second most common neoplasm found in the head and neck region. However, isolated lymphomas of the head and neck region may present diagnostic difficulties, as the clinical presentation is occult and atypical. There is often difficulty in establishing definitive tissue diagnosis with core needle biopsies. There is a limited standard guideline as to the treatment of choice for this pathology, and the role of surgery differs on a case-by-case basis. We present the case of an adult male who presented with neck pain and spasms and was found to have a spinal diffuse large B-cell lymphoma (DLBCL), a double expressor phenotype that extended from retropharyngeal space to the cervical spine.

## Case presentation

**Figure 1:**
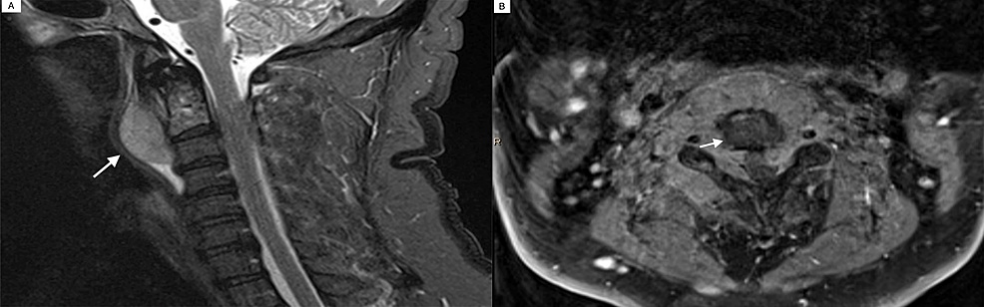
Magnetic resonance imaging (MRI) of the mass lesion A: sagittal T2 showed a retropharyngeal mass at the level of C2 extending to the C3 level. B: axial T1 postcontrast showed moderate to severe central canal narrowing.

A 58-year-old male presented to the neurosurgery spine clinic with a one-year history of chronic neck pain and spasm. Past medical history was significant for malignant gastrointestinal mucosal melanoma treated via surgical resection with lymph nodes negative for metastasis, obstructive sleep apnea, hypertension, and hyperlipidemia. On routine workup for neck pain and spasm, C-spine imaging was obtained, showing an avidly enhancing retropharyngeal mass extending through the bilateral neuroforamina, into the epidural space, and involving the posterior elements of the cervical spine at C2-C3. The mass measured 1.8 x 4.7 x 4.5 cm (AP x SI x RL) with associated moderate to severe central canal narrowing at the C2 extending to the C3 level with the deformation of the spinal cord (Figure 1, A and B). There was no abnormal spinal cord signal or enhancement. Computed tomography (CT) imaging of the chest, abdomen, and pelvis did not indicate any metastatic disease or another primary mass lesion. Physical examination revealed no focal neurological deficit and no positive findings except neck pain and subjective dysphagia.

**Figure 2:**
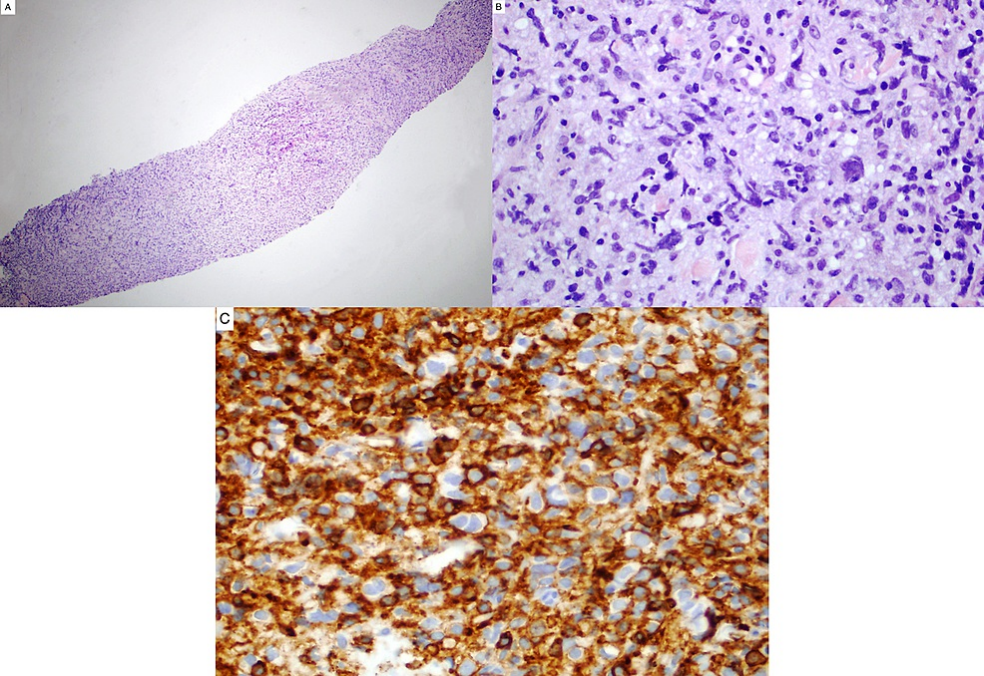
Needle core biopsy of the retropharyngeal mass lesion A: microscopically, at low magnification, hematoxylin and eosin (H&E) stain showed highly cellular neoplasm. B: at higher power, H&E stain showed small to medium size cells in the fibrous background with large atypical epithelioid cells. C: CD45 was strongly positive in an obscuring background benign lymphocytes with some atypical cells negative.

Despite the somewhat benign clinical presentation, the concerning imaging findings prompted a subsequent CT-guided needle-core biopsy. This biopsy was limited by tissue quantity and artifacts but demonstrated the presence of a malignant neoplasm of undetermined type, composed of rare large atypical epithelioid cells with prominent nucleoli in a crushed inflammatory and fibrous background. Mitotic figures were easily found with no necrosis identified. There was no distinctive glandular architecture (Figure 2, A and B). Immunohistochemistry showed no immunoreactivity in the atypical cells for cytokeratin (AE1/AE3 and CAM5.2) and melanocyte markers (HMB45, S100 protein, Melan-A/Mart-1). CD45 evaluation was obscured by many background benign lymphoid cells, with some of the atypical cells showing potential reactivity (Figure 2, C). The overall appearance of the neoplastic cells was not suggestive of lymphoma.

This pathologic finding prompted the need for partial excision to yield more tissue for definitive diagnosis. Without knowing the pathologic nature of the mass, it is difficult to adequately treat this kind of lesion. Given a CT-guided biopsy only yielded that the mass was neoplasm, we had a choice between attempting gross total resection of the mass or open biopsy. On the other hand, attempting gross total resection would have entailed an anterior approach (transoral with possible odontoidectomy or endoscopic endonasal with possible odontoidectomy) along with posterior instrumentation and fusion from occiput to C3, which is a rather morbid procedure that subjects the patient to a decreased quality of life as well as risks of vascular injury, dysphagia, and infection.

We elected to perform an open biopsy of the epidural component of the mass through a posterior decompressive laminectomy, which allowed for decompression of the spinal cord as well as a sampling of the mass. This provided treatment for possible increasing epidural compression from the mass, as well as diagnostic tissue. The open biopsy did yield a result that was treatable by chemoradiation options. The patient tolerated the procedure well and experienced significant improvement in his neck pain shortly after.

**Figure 3:**
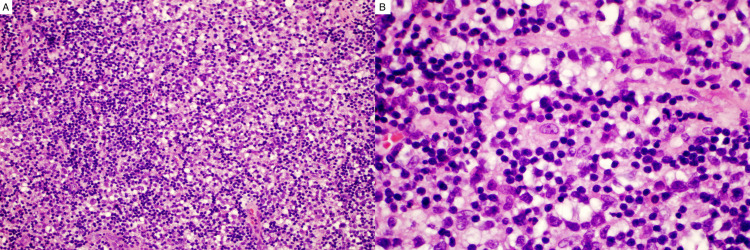
Epidural mass lesion A: microscopically, at low magnification (200x), the hematoxylin and eosin (H&E) stain showed a highly cellular lymphoid mass with small benign reactive appearing lymphocytes. B: at higher power (600x), small lymphocytes and large atypical cells with prominent nucleoli and large cytoplasm were seen.

Histologic and immunohistochemical studies of the second biopsy revealed double expressor DLBCL with anaplastic features. The lymphoma was present only in the epidural soft tissue and was not recognized in or on the specimens of bone. At low power, the field showed mostly small benign, reactive appearing lymphocytes (Figure 3, A). At higher power, there were small lymphocytes and large atypical cells with prominent nucleoli and large cytoplasm (Figure 3, B).

This unusual hematoxylin and eosin (H&E) appearance (atypical cells with prominent nucleoli and large cytoplasm) of the tumor suggested a differential diagnosis of lymphoma, germ cell neoplasm (germinoma), clear cell renal cell carcinoma, melanoma, and liposarcoma.

Immunostains were negative for characteristic markers of many of the tumors in the differential diagnosis, including those for germ cell tumors (OCT3/4-, PLAP-), melanoma (S100-, SOX10-), liposarcoma (S100-), and renal cell carcinoma (PAX8-). Keratins and epithelial membrane antigen (EMA) were also negative, excluding carcinomas.

There was strong CD45 immunoreactivity, in small appearing lymphocytes and the larger vacuolated atypical cells establishing a diagnosis of lymphoma, with a differential of B-cell lymphomas, T-cell lymphomas, and Hodgkin's lymphomas.

**Figure 4:**
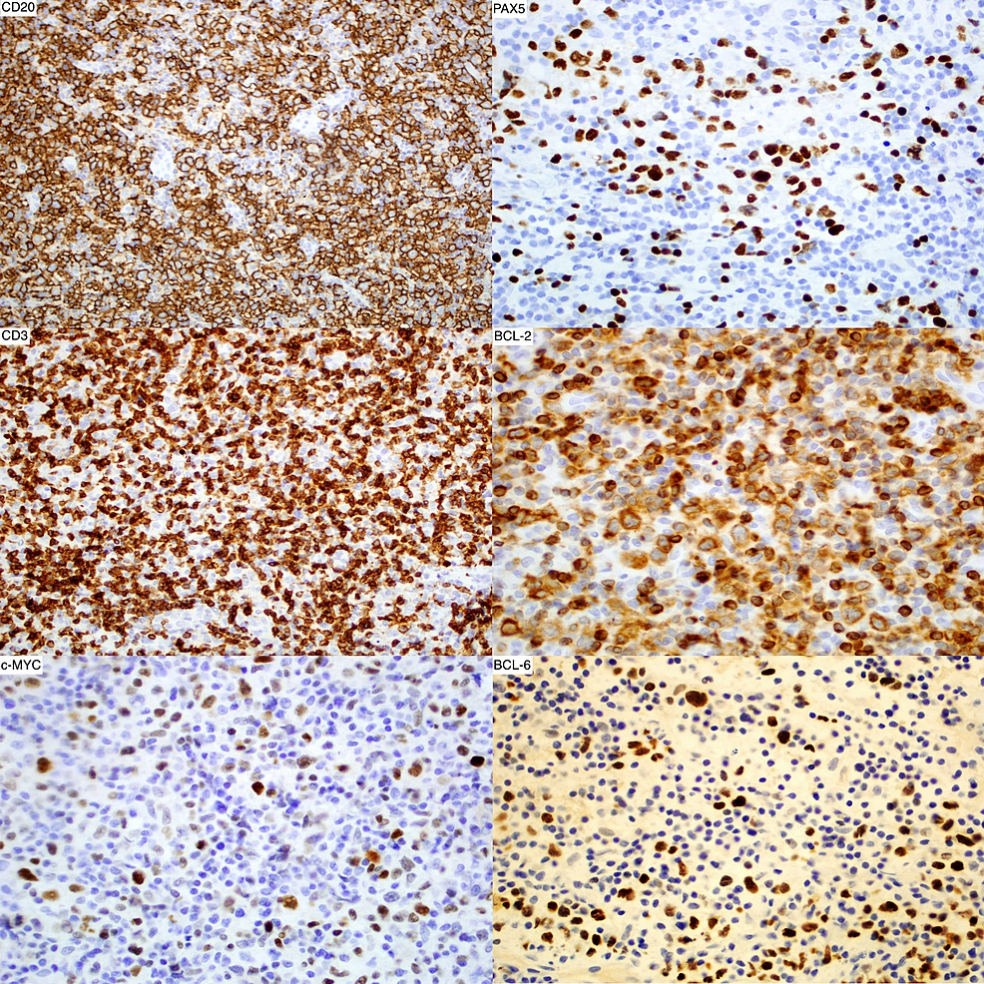
Immunohistochemistry stain showed the immunopositivity of the atypical neoplastic cells for CD20 and PAX5. The large atypical cells were also positive for CD20, PAX5, BCL-6, and c-MYC.

The tumor had numerous infiltrating small reactive T lymphocytes, which were CD3, CD5, CD43, and B-cell lymphoma-2 (BCL-2) immunopositive, but the larger atypical cells were only positive for BCL-2 (Figure 4). The large atypical cells were also positive for CD20 and Pax5 (Figure 4). The majority of B-cell malignancies, including NHLs, are immunopositive for both CD20 and PAX5. A subset of these large atypical cells was immunopositive for CD10, and many were immunopositive for BCL-6 and c-MYC (Figure 4). The tumor has no immunoreactivity for CD30, and only a few cells are immunopositive for CD15, ruling out Hodgkin's lymphoma. The neoplastic cells were also positive for cyclin D1. 

MUM-1 and SOX11 immunostains were negative, and a Ki67 revealed a substantial level of proliferative activity, but a labeling index is essentially impossible to determine given a large number of non-neoplastic T cells in the lesion. 

These immunophenotypes established the diagnosis of B-cell lymphoma, which then histologically was a diffuse large B cell type. The CD10 immunoreactivity identified the germinal center phenotype. The cyclin D1 immunopositivity, BCL-2 immunopositivity, and c-MYC immunoreactivity suggested an anaplastic component.

The Bcl-2 plus c-MYC immunoreactivity represented the foundation for the "double expressor" phenotype, which prompted molecular genetic analysis by fluorescence in situ hybridization (FISH) to determine if this is a double-hit lymphoma. Analysis of the tumor DNA using a variety of FISH strategies (including use of "break-apart probes" and dual-color FISH) indicated that there was a rearrangement of the BCL-2 gene (found in about 35% of the tumor cell nuclei), but no BCL-6 gene or MYC gene rearrangement was detected. There was also no fusion gene formed from MYC and the immunoglobulin heavy chain (IGH) gene. These results indicated that the lymphoma cannot be characterized as a double-hit high-grade B-cell lymphoma with c-MYC and BCL-2 rearrangements since only the BCL-2 gene abnormal rearrangement was detected. However, the immunoreactivity for BCL-2 and c-MYC established the diagnosis of double-expressor diffuse large cell B-cell non-Hodgkin's lymphoma with focal anaplastic features. These immunophenotypes are considered poor prognostic indicators.

Surgical resection (transoral with possible odontoidectomy or endoscopic endonasal with possible odontoidectomy) is usually considered a default option for the management of this kind of tumor. In order to access the C2-C3 region through the endoscopic endonasal approach, the hard palate would most likely need to be split. Performing an odontoidectomy and dissecting laterally enough to remove the mass where it enters the central canal could pose significant injury to the nearby vertebral arteries. Moreover, these approaches (endoscopic endonasal or transoral) tend to introduce significant morbidity and complications to the patient, including possible communicating infection, limited resection window, and postoperative dysphagia. Since the patient presented with only neck pain and spasms without clinical signs of spinal instability or cord compression, a judicious decision had to be made when the pathology of the tumor was unclear. These factors prompted discussion and collaboration of care among the multidisciplinary panel, including radiation oncology and hematology-oncology team, to develop a treatment plan. 

A multidisciplinary panel came together to discuss the case and developed a treatment plan for the patient with systemic and intrathecal chemotherapy in combination with radiotherapy. At a recent follow-up, the patient was doing well with no neurological symptoms.

## Discussion

Non-Hodgkin's lymphoma (NHL) is a malignancy of lymphoid tissues and can originate from B cells and T cells (both precursors and mature cells). NHL can involve any site in the extracranial head and neck, and it is the second most common malignancy of the head and neck region, behind squamous cell carcinoma [1]. It accounts for 85% of spinal lymphoma cases. DLBCL is the most common subtype of aggressive NHL that originates from the germinal center and has differential molecular and clinical features [2]. Although any segment of the spine can be affected by NHL, it usually affects the thoracic spine [3,4]. Spinal lymphomas have been reported in all age groups, but patients clinically present most commonly in the fifth to seventh decades of life with a 16:1 male to female ratio [5].

Non-Hodgkin's lymphomas rarely involve the spine, only in about 0.1% to 6.5% of cases [5]. Spinal lymphomas comprise less than 2% of all lymphomas and make up about 10% of all neoplasms that involve epidural space [4,6]. The tumorigenesis of spinal lymphoma is not fully understood. It is thought that lymphomas first originate from adjacent vertebrae or paraspinal soft tissues such as epidural lymphoid tissues and then locally invade the epidural space through the vertebral foramen without causing erosion of the bone [7].

Clinical presentation of NHL varies based on the subtypes and aggressiveness of the lymphoma. Diffuse large B-cell lymphomas usually present as rapidly enlarging mass or lymphadenopathy with associated constitutional symptoms and are considered clinically aggressive. When they have epidural space involvement, they can present with cord compression symptoms [6].

Several molecular alterations, such as c-MYC (codes for transcription factor), BCL-2 (oncogene), and BCL-6 (transcription repressor), have been identified in the pathogenesis of subtypes of DLBCL and have prognostic implications [8]. These genes are involved in cellular transcription, survival, proliferation, and apoptosis. Simultaneous rearrangement of c-MYC, BCL2, and BCL6 genes are involved in double-hit lymphomas, which are high-grade B cell lymphomas with poor prognosis [2,4]. DLBCL is considered a double expressor lymphoma when overexpression involves two of these genes without gene rearrangement or translocations [4,8,9].

When approaching a spinal lymphoma case, it is important to localize the tumor with imaging studies to identify if the lymphoma is a primary lesion or represents the metastatic spread of systemic lymphoma. The typical presentation of spinal lymphoma is a T1 isointense or hypointense homogeneous mass on MRI in the epidural space that involves multiple segments, enhances with contrast, and can have foraminal extension [10]. A CT-guided biopsy is considered the next approach to diagnose lymphoma when imaging findings are suggestive. Needle biopsy, however, often fails to achieve a diagnostic sample [11]. This fact is highlighted in our case, where the initial CT-guided biopsy did not yield a representative sample and was inconclusive. The diagnosis was only established through an open biopsy of the mass.

There is a limited standard guideline for the treatment of spinal DLBCL, but various modalities have been proposed. The proposed therapeutic regiments include a combination of radiotherapy and chemotherapy, radiation alone, or surgical management combined with radiotherapy and chemotherapy [12]. Several factors also play a role in choosing appropriate therapeutic modalities, including clinical information (patient's age, stage, and subtype of the DLBCL), molecular features, and the presence or absence of spinal cord compression symptoms [13]. The molecular feature of the spinal lymphoma determines the prognosis and response to therapy. Several studies have reported the poor prognostic implication of double expressor lymphoma when compared to DLBCL without this phenotype [8].

The chemotherapy modalities that are currently being utilized include etoposide, prednisone, vincristine, cyclophosphamide, doxorubicin, rituximab (EPOCH-R), rituximab, cyclophosphamide, doxorubicin, vincristine, prednisone (R-CHOP), and other intensive regiments. Several novel chemotherapy and immunotherapy agents and adjunct stem cell transplant are also currently under clinical trials [2,8].

The literature offers conflicting evidence on the role of primary surgical decompression, as lymphoma historically has a good response to radiotherapy and chemotherapy. However, in patients with cord compression symptoms, surgical excision is recommended for decompression and would also obtain tissue for excisional biopsy to establish the diagnosis [14]. Chang et al. reported positive survival benefits of surgical decompression in patients diagnosed with DLBCL of the spine that presented with spinal cord compression features [15]. On the other hand, Peng et al. reported negative survival outcomes of surgical decompression alone compared to a combination of radiotherapy and chemotherapy followed by decompression surgery in patients with malignancy-associated spinal cord compression [12]. Despite the lack of standard treatment guidelines for spinal lymphomas, a multidisciplinary approach should be considered in developing an effective and patient-centered management plan for patients with spinal DLBCL [16].

Regarding the prognosis, double expressor lymphoma of the spine is generally considered aggressive and has a poor prognosis, especially in elderly patients [14, 17]. Five-year relative survival for patients with DLBCL is about 63% for all ages [18].

## Conclusions

Our patient, a 58-year-old male, presented with neck pain and spasm and was diagnosed with primary diffuse large B-cell lymphoma of the spine, a double expressor phenotype. The diagnosis of this entity was complicated by the delay in obtaining a satisfactory tissue sample. The heterogeneity of the modalities of treatment tailored to the particular patient’s situation, in addition to taking into consideration the patient's surgical outcome and quality of life, played an extensive part in the decision-making in this case. For a dumbbell tumor high in the cervical region, with an inconclusive biopsy, it is a reasonable option to perform laminectomy and open biopsy rather than proceeding with a definitive attempt at gross total resection, hoping that the biopsy will yield a pathology that is treatable with chemoradiation, thus absolving the need to subject the patient to a risky procedure with significant complications. Our patient underwent posterior decompression and excisional biopsy without resection of the tumor. Following surgical intervention, the patient experienced significant improvement in his neck pain. The patient is currently doing well and being followed by a multidisciplinary team from radiation oncology, hematology-oncology, and neurological surgery.

## References

[1] Hermans R, Horvath M, De Schrijver T, Lemahieu SF, Baert AL (1994). Extra nodal non-Hodgkin lymphoma of the head and neck. J Belge Radiol.

[2] Liu Y, Barta SK (2019). Diffuse large B-cell lymphoma: 2019 update on diagnosis, risk stratification, and treatment. Am J Hematol.

[3] Simiele Narvarte A, Gómez Rodríguez N, Novoa Sanjurjo F (2003). Compromiso epidural espinal como presentación de los linfomas no hodgkinianos: aportación de 6 casos. (Article in Spanish). An Med Interna.

[4] Mufti M, Nawab K, Mohammad R (2018). A great mimicker in thoracic spine: spinal double expressor lymphoma. J Hematol.

[5] Salvati M, Cervoni L, Artico M, Raco A, Ciappetta P, Delfini R (1996). Primary spinal epidural non-Hodgkin's lymphomas: a clinical study. Surg Neurol.

[6] Perry JR, Deodhare SS, Bilbao JM, Murray D, Muller P (1993). The significance of spinal cord compression as the initial manifestation of lymphoma. Neurosurgery.

[7] Seo JY, Ha KY, Kim MU, Kim YC, Kim YH (2014). Spinal cord compression by B-cell lymphoma, unclassifiable, with features intermediate between diffuse large B-cell lymphoma and Burkitt lymphoma in a patient seropositive for human immunodeficiency virus: a case report. J Med Case Rep.

[8] Hu S, Xu-Monette ZY, Tzankov A (2013). MYC/BCL2 protein coexpression contributes to the inferior survival of activated B-cell subtype of diffuse large B-cell lymphoma and demonstrates high-risk gene expression signatures: a report from The International DLBCL Rituximab-CHOP Consortium Program. Blood.

[9] Riedell PA, Smith SM (2018). Double hit and double expressors in lymphoma: definition and treatment. Cancer.

[10] Mascalchi M, Torselli P, Falaschi F, Dal Pozzo G (1995). MRI of spinal epidural lymphoma. Neuroradiology.

[11] Haque S, Law M, Abrey LE, Young RJ (2008). Imaging of lymphoma of the central nervous system, spine, and orbit. Radiol Clin North Am.

[12] Peng X, Wan Y, Chen Y (2009). Primary non-Hodgkin's lymphoma of the spine with neurologic compression treated by radiotherapy and chemotherapy alone or combined with surgical decompression. Oncol Rep.

[13] Cho HJ, Lee JB, Hur JW, Jin SW, Cho TH, Park JY (2015). A rare case of malignant lymphoma occurred at spinal epidural space: a case report. Korean J Spine.

[14] Mally R, Sharma M, Khan S, Velho V (2011). Primary lumbo-sacral spinal epidural non-Hodgkin's lymphoma: a case report and review of literature. Asian Spine J.

[15] Chang CM, Chen HC, Yang Y, Wang RC, Hwang WL, Teng CL (2013). Surgical decompression improves recovery from neurological deficit and may provide a survival benefit in patients with diffuse large B-cell lymphoma-associated spinal cord compression: a case-series study. World J Surg Oncol.

[16] Tang Y, Yang X, Xiao J (2013). Clinical outcomes of treatment for spinal cord compression due to primary non-Hodgkin lymphoma. Spine J.

[17] Hashi S, Goodwin CR, Ahmed AK, Sciubba DM (2018). Management of extranodal lymphoma of the spine: a study of 30 patients. CNS Oncol.

[18] Martelli M, Ferreri AJ, Agostinelli C, Di Rocco A, Pfreundschuh M, Pileri SA (2013). Diffuse large B-cell lymphoma. Crit Rev Oncol Hematol.

